# Entlassbarkeit versus Entlassung – Ergebnisse der PROMISE-Studie

**DOI:** 10.1007/s00132-022-04247-4

**Published:** 2022-04-12

**Authors:** Ulrich Betz, Michael Clarius, Manfred Krieger, Laura Langanki, Matthias Büttner, Sabine Fencel, Lukas Eckhard, Thomas Klonschinski, Philipp Drees

**Affiliations:** 1 Institut für Physikalische Therapie, Prävention und Rehabilitation, Universitätsmedizin Mainz, Langenbeckstr. 1, 55131 Mainz, Deutschland; 2 Vulpius Klinik GmbH, Bad Rappenau, Deutschland; 3 Abteilung Orthopädische Chirurgie, Klinikum GPR Rüsselsheim, Rüsselsheim, Deutschland; 4 Institut für Medizinische Biometrie, Epidemiologie und Informatik, Universitätsmedizin, Mainz, Deutschland; 5 Zentrum für Orthopädie und Traumatologie, Universitätsmedizin, Mainz, Deutschland

**Keywords:** Enhanced Recovery after Surgery, Hüftendoprothetik, Knieendoprothetik, Hospitalisierungsdauer, Patientenentlassung, Enhanced Recovery after Surgery, Hip replacement, Hospital stay, Knee replacement, Patient discharge

## Abstract

**Hintergrund:**

Für Deutschland werden im Rahmen optimierter Behandlungsprozesse rund um die Implantation einer Knie- oder Hüftendoprothese stationäre Aufenthaltszeiten angegeben, die die international publizierten Zeiten deutlich übertreffen. Die vorliegende Analyse von Daten der PROMISE-Studie beschäftigt sich in diesem Zusammenhang mit dem Verhältnis von Entlassbarkeit und Entlassung.

**Methode:**

In drei Krankenhäusern unterschiedlicher Versorgungsstufen wurde ein gemeinsam entwickelter, optimierter Versorgungsstandard etabliert und für eine weitgehend unselektionierte Patientenkohorte umgesetzt. Unter anderem wurden Daten zum Erreichen der Entlasskriterien (EK) und der tatsächlichen Entlassung erhoben. Univariate Vergleiche wurden durch Chi-Quadrat-Tests oder Mann-Whitney-U-Tests durchgeführt.

**Ergebnisse:**

Insgesamt konnten 1782 Patienten eingeschlossen werden, von denen 85,3 % nach im Mittel (MW) 2,4 Tagen (Median 2) postoperativ alle zuvor definierten EK erreicht haben. Die Entlassung für diese Gruppe erfolgte nach 5,4 (MW) Tagen (Median 5). Die restlichen Probanden (14,7 %) hatten bei Entlassung nach 6,5 (MW) Tagen (Median 6) mindestens ein EK nicht erreicht. Für verschiedene Subgruppen konnten signifikante Unterschiede festgestellt werden.

**Fazit:**

Die sogenannten EK werden als relative Kriterien eingesetzt. Das Erreichen führt im Allgemeinen nicht zu einer zeitnahen Entlassung der Patienten aus dem Krankenhaus. Wäre dies der Fall, wären auch in Deutschland international übliche Aufenthaltszeiten Realität. Was die Entlassung tatsächlich bestimmt, bleibt offen. Eine Vielzahl von medizinischen, organisatorischen, strukturellen und finanziellen Einflussfaktoren könnten bedeutend sein.

## Abstract (en)

**Background:**

In the context of optimized treatment processes for knee and hip replacements, lengths of stay are given for Germany that clearly exceed the internationally published ones. In this context, the present analysis of data from the PROMISE study deals with the relationship between discharge readiness and discharge.

**Methods:**

In the PROMISE study, a jointly developed, optimized standard of care was established in three hospitals of different levels of care and realized for a typical, largely unselected cohort of patients. Among others, data were collected on achievement of discharge criteria (DC) and actual discharge. Univariate comparisons were performed by chi-square tests or the Mann-Whitney‑U tests.

**Results:**

A total of 1782 patients were included, of whom a mean of 85.3% achieved all previously defined DCs at a mean of 2.4 (median 2) days postoperatively. Discharge for this group occurred after a mean of 5.4 (median 5) days. 14.7% of the participants did not achieve at least one DC. This group was discharged after a mean of 6.5 (median 6) days. Significant differences in outcomes were observed for different subgroups.

**Conclusion:**

The so-called DCs are used as relative criteria. Achievement generally does not result in timely discharge. If this were to happen, internationally established lengths of stay would also be a reality in Germany. What actually determines discharge from inpatient treatment remains open. A variety of medical, organizational, structural and financial factors could be of importance.

International werden für Patienten in der Hüft- und Knieendoprothetik, die einen optimierten Behandlungsprozess nach dem Konzept der Enhanced Recovery after Surgery (ERAS) durchlaufen, postoperativ deutlich kürzere stationäre Aufenthaltszeiten genannt als in Deutschland. Werden die Entlasskriterien hier später erreicht, oder bleiben die Patienten stationär, obwohl die Entlasskriterien bereits erreicht sind? Zu diesem Fragenkomplex wurden im Rahmen der PROMISE-Studie Daten erhoben, die nachfolgend vorgestellt werden.

## Hintergrund und Fragestellung

Die Prinzipien des Enhanced Recovery after Surgery (ERAS) auch in der Hüft- und Knieendoprothetik anzuwenden, ist international inzwischen weit verbreitet. Die ERAS Society hat für die Durchführung ein Konsenspapier veröffentlicht [1, 2] und es liegen mehrere systematische Reviews und Metaanalysen zu den Behandlungsresultaten vor [3–5]. Als durchschnittliche stationäre Aufenthaltsdauer werden international 2–4 Tage genannt [6–9]. Für vorselektionierte Gruppen sind auch stationäre Aufenthaltszeiten unter 24 h möglich [10]. Auch in Deutschland findet das ERAS-Konzept in der Endoprothetik zunehmend Anwendung [11]. Für eine ausgewählte Patientengruppe im Bereich der Hüftendoprothetik wird hier ebenfalls von stationären Aufenthaltszeiten < 24 h berichtet [12]. Für unselektionierte Kohorten werden in Deutschland 5–7 Tage bis zur Entlassung aus dem Krankenhaus angegeben [13, 14].

Sind Entlasskriterien angegeben, unterscheiden sich diese nicht grundsätzlich. Gefordert werden ein Mindestmaß an selbstständiger Aktivität (Lagewechsel, Essen am Tisch, Anziehen, Waschen, Mindestgehstrecke, Treppensteigen), tolerierbarer Schmerz und trockene Wundverhältnisse [8, 13–16]. Bei „hip in a day“ sind die Entlasskriterien noch um die Abwesenheit von Schwindel, Übelkeit, Erbrechen oder Schwäche und keine Einwände durch Operateur, Anästhesist oder Pflege erweitert. Zudem muss der Patient einen Coach an seiner Seite haben [12].

Holm et al. [8] geben für Patienten nach Implantation einer Hüftendoprothese in Dänemark das Erreichen der Entlasskriterien im Mittel mit 1,9 Tagen (Knie-TEP 2,3 Tage) und die Entlassung aus stationärer Behandlung mit 2,0 Tagen (Knie-TEP 3,0 Tage) an. Vergleichbare Daten für Deutschland sind nach bestem Wissen der Autoren bisher nicht veröffentlicht. Dieses Defizit kann nun mit Ergebnissen der PROMISE-Studie (Prozessoptimierung durch interdisziplinäre und sektorenübergreifende Versorgung am Beispiel von Patienten mit Hüft- und Knieendoprothesen; [17]) behoben werden. Die prospektive, multizentrische Studie hat untersucht, ob durch einen optimierten Versorgungsprozess im Sinne des Enhanced Recovery die Qualität in der operativen Versorgung von Patienten mit Hüft- und Kniegelenkarthosen in Deutschland nachhaltig gesteigert werden kann. Dabei wurden Daten für das Erreichen der Entlasskriterien, hier genannt Entlassbarkeit, und die tatsächliche Entlassung gewonnen, die nachfolgend vorgestellt werden.

## Studiendesign und Untersuchungsmethoden

Drei Krankenhäuser unterschiedlicher Versorgungsstufen, ein Belegbettenhaus, ein orthopädisches Fachkrankenhaus und ein Universitätsklinikum, wurden mit allen erforderlichen klinischen Fachabteilungen in die PROMISE-Studie einbezogen. Um einen intersektoralen Behandlungsbruch zu vermeiden, wurden zusätzlich fünf Rehabilitationseinrichtungen (vier stationäre und eine ambulante) als Kooperationspartner integriert.

Ab März 2017 definierte ein interdisziplinäres Gremium, bestehend aus Experten aller beteiligten Professionen und Einrichtungen, einen gemeinsamen optimierten Versorgungsstandard. Im Anschluss wurde das Protokoll in den verschiedenen Einrichtungen gleichermaßen etabliert und konsolidiert.

Im Kern besteht der optimierte PROMISE-Prozess aus evidenzbasierten Interventionen, die mittlerweile von der ERAS Society für die Behandlung in der Hüft- und Knieendoprothetik empfohlen werden [1, 2]. Lediglich präoperative Optimierungsempfehlungen zur Raucherentwöhnung für mindestens 4 Wochen vor der Operation und Alkoholentwöhnungsprogramme für Alkoholabhängige waren nicht Teil der Studie. Diese beiden Komponenten konnten nicht nachträglich in das Studienprotokoll aufgenommen werden, da der Einschluss der Patienten zum Zeitpunkt der Veröffentlichung der Empfehlungen bereits weit fortgeschritten war.

Die von ERAS empfohlenen Maßnahmen werden durch Interventionen ergänzt, die den Prozess weiter unterstützen (Patient definiert ein spezifisches Aktivitäts‑/Partizipationsziel; Patient hat einen Coach, eine persönliche Vertrauensperson, die ihn durch den Prozess begleitet; Patientenmanagement und Sozialdienste sind in den Prozess eingebunden) und die Mobilitätsbarrieren reduzieren (kein Harnkatheter, keine pneumatische Blutsperre [Knie-TEP], keine Drainagen). Um die möglichen Risiken schlechter Ergebnisse zu verringern, wurde ein Screening bezüglich geriatrischer (ISAR-Screening [18]) und psychosomatischer Risiken (Patient Health Questionnaire‑4 [19]) durchgeführt.

Voraussetzungen für den Einschluss waren, dass die Patienten die standardisierten Kriterien für eine Hüft-TEP oder Knie-TEP erfüllen [20] und in der Lage waren, Art und Umfang der Studie zu verstehen.

Ausschlusskriterien waren eine Lebenserwartung von weniger als einem Jahr (z. B. Krebs im fortgeschrittenen Stadium), Erkrankungen, die einen elektiven chirurgischen Eingriff ausschließen könnten sowie medizinische oder psychologische Faktoren, die eine Teilnahme oder eine schriftliche Einwilligung nach Aufklärung verhindern würden.

Das Studienprotokoll wurde ausführlich beschrieben [17] und durch die Ethikkommissionen in Rheinland-Pfalz (837.533.17 [11367]), Baden-Württemberg (B-F-2018-042) und Hessen (MC 84/2018) freigegeben. Die Studie ist registriert im Deutschen Register für Klinische Studien (DRKS00013972) und wurde finanziert vom Innovationsfond des gemeinsamen Bundesausschusses (01NVF16015).

Für diese Studie wurden folgende Entlasskriterien definiert: selbstständiger Lagewechsel (Liegen, Sitzen, Stehen), selbstständiges Essen und Trinken am Tisch, selbstständige Körperpflege, selbstständiges An- und Entkleiden, Schuhe anziehen, selbstständig > 150 m gehen, Treppen steigen (10 Stufen), unauffällige Wundverhältnisse, tolerierbarer Schmerz (NRS ≤ 5). Wurden die Kriterien erreicht, wurde der Zeitpunkt in einer elektronischen Datenbank festgehalten. Wurde das Kriterium nicht erreicht, wurde ein Freitext erfasst.

Deskriptive Beschreibungen wurden mittels Mittelwerten, Medianen und Anteilen dargestellt. Univariate Vergleiche wurden, in Abhängigkeit der Daten, durch Chi-Quadrat-Tests oder den Mann-Whitney-U-Test durchgeführt.

## Ergebnisse

### Probandenkohorte

Insgesamt kann zur Analyse auf 1782 Datensätze von Patienten zurückgegriffen werden, bei denen aufgrund von Hüft- oder Kniearthrose zwischen Mai 2018 und März 2020 ein Gelenkersatz implantiert wurde. Die Patienten waren im Durchschnitt 67 Jahre alt, mit einem geringen Überhang an weiblichen Teilnehmerinnen (56 %). Hüft- und Kniepatienten waren jeweils zu 50 % vertreten. In der ASA-Klassifikation („grading of patients for surgical procedures“ von der American Society of Anesthesiologists) dominierte mit 63 % Grad 2 (Patient mit leichter Allgemeinerkrankung). Die postoperative stationäre Aufenthaltszeit betrug im Mittel 5,4 Tage. Eine detaillierte Beschreibung der Probandengruppe ist in Tab. 1 dargestellt.

**Table 1:** 

	Gesamt	Alle Kriterien erfüllt	Nicht alle Kriterien erfüllt
*Anzahl n (Anteil)*	1782 (100 %)	1520 (85,3 %)	262 (14,7 %)
*Aufenthalt Tage (p-Wert *< 0,001)			
Mittelwert (SD)	5,4 (3,3)	5,2 (2,7)	6,5 (5,5)
Median (Q1; Q3)	5 (4; 6)	5 (4; 6)	6 (5; 7)
Spannweite	0–84	0–37	0–84
*Alter (p-Wert *0,0503)			
Mittelwert (SD)	66,5 (10,1)	66,4 (10,0)	67,6 (10,8)
Median (Q1; Q3)	67 (60; 74)	67 (59; 74)	69 (60; 76)
Spannweite	23–93	23–93	23–91
*Geschlecht (p-Wert 0,002)*			
Männlich	44,2 % (788)	45,8 % (696)	35,1 % (92)
Weiblich	55,8 % (994)	54,2 % (824)	64,9 % (170)
*Lokalisation (p-Wert *< 0,001)			
Knie	49,6 % (882)	47,1 % (716)	63,6 % (166)
Hüfte	50,4 % (898)	52,9 % (803)	36,4 % (95)
*ASA (p-Wert *< 0,001)			
1	6,5 % (115)	6,8 % (102)	5,0 % (13)
2	62,5 % (1098)	64,7 % (970)	49,4 % (128)
3	30,4 % (534)	28,2 % (422)	43,2 % (112)
4	0,6 % (11)	0,3 % (5)	2,3 % (6)

Der angegebene p‑Wert bezieht sich auf die Unterschiede zwischen den Gruppen EKe und EKn

*ASA* American Society of Anesthesiologists

### Nicht alle Patienten erreichen alle Entlasskriterien

Von 85,3 % (*n* = 1520) aller Teilnehmenden konnten während des stationären Aufenthaltes alle Entlasskriterien erreicht werden (Gruppe EKe). In der Frage ob die Entlasskriterien erreicht wurden oder nicht, spielt das Alter keine relevante Rolle. Der Anteil der weiblichen Patientinnen ist in der Gruppe derjenigen, die die Entlasskriterien nicht komplett erreichen (Gruppe EKn) konnten, gegenüber der Gruppe EKe signifikant größer (EKe 54,2 %; EKn 64,9 %; *p* = 0,002). Ebenso verhält es sich mit den Kniepatienten (EKe 47,1 %; EKn 63,6 %; *p* < 0,001). Der Anteil der ASA-2-Patienten dominiert auch in der Gruppe EKn (49,4 %), aber der Anteil der ASA-3-Patienten ist auf 43,2 % gegenüber EKe (28 %) erhöht (*p* < 0,001). Die postoperative Aufenthaltszeit unterscheidet sich zwischen den Gruppen EKe (5,2 Tage) und EKn (6,5 Tage) ebenfalls signifikant (*p* < 0,001) (Tab. 1).

### Wann werden die Entlasskriterien erreicht?

In der Gruppe EKe (85,3 %; *n* = 1520), dauert es im Mittel 2,4 Tage (SD 1,9) bis das letzte Kriterium erreicht ist (Median [Q1; Q3]: 2 [2; 3]; Spannweite: 0–29), Hüftpatienten benötigen im Mittel 2,2 Tage (SD 1,8) (Median [Q1; Q3]: 2 [1; 2]; Spannweite: 0–29) und Kniepatienten im Mittel 2,6 Tage (SD1,9) (Median [Q1; Q3]: 2 [2; 3]; Spannweite: 0–23).

Damit sind in dieser Gruppe die Entlasskriterien im Mittel nach 47 % (SD 22 %) (Median [Q1; Q3]: 40 % [33 %;60 %], Spannweite: 0–100 %) der Aufenthaltszeit erreicht, Kniepatienten benötigen im Mittel 49 % der Aufenthaltszeit (SD 21 %) (Median [Q1; Q3]: 50 % [38 %;60 %]; Spannweite: 0–100 %) und Hüftpatienten im Mittel 45 % der Aufenthaltszeit (SD 22 %) (Median [Q1; Q3]: 40 % [25 %;60 %]; Spannweite: 0–100 %) bis zum Erreichen des letzten Entlasskriteriums.

In der Gesamtgruppe werden die Kriterien zu hohen, aber unterschiedlichen Prozentzahlen erreicht. Folgende Kriterien werden von weniger als 98 % der Patienten erreicht: selbstständiges Essen und Trinken am Tisch (Knie 96,7 %; Hüfte: 97,5 %), selbstständig > 150 m gehen (Knie: 95,7 %; Hüfte: 96,7 %), Treppen steigen (Knie: 95,1 %; Hüfte: 95,9 %), tolerierbarer Schmerz (Knie 93 %). Das Kriterium „tolerierbarer Schmerz (Knie)“ ist das am häufigsten bis zur Entlassung nicht erreichte Kriterium (7 %).

Die verschiedenen Entlasskriterien werden im Mittel in folgenden postoperativen Zeitabschnitten erreicht:

Tag 0–1: selbstständiger Lagewechsel (Liegen, Sitzen, Stehen) (Knie und Hüfte), selbstständiges Essen und Trinken am Tisch (Hüfte), selbstständige Körperpflege (Knie und Hüfte), selbstständiges An- und Entkleiden (Knie und Hüfte), Schuhe anziehen (Knie und Hüfte), unauffällige Wundverhältnisse (Knie und Hüfte).

Tag 1–2: selbstständiges Essen und Trinken am Tisch (Knie), selbstständig > 150 m gehen (Knie und Hüfte), Treppen steigen (Hüfte), tolerierbarer Schmerz (Knie und Hüfte).

> 2 Tage: Treppen steigen (Knie).

**Figure 1:**
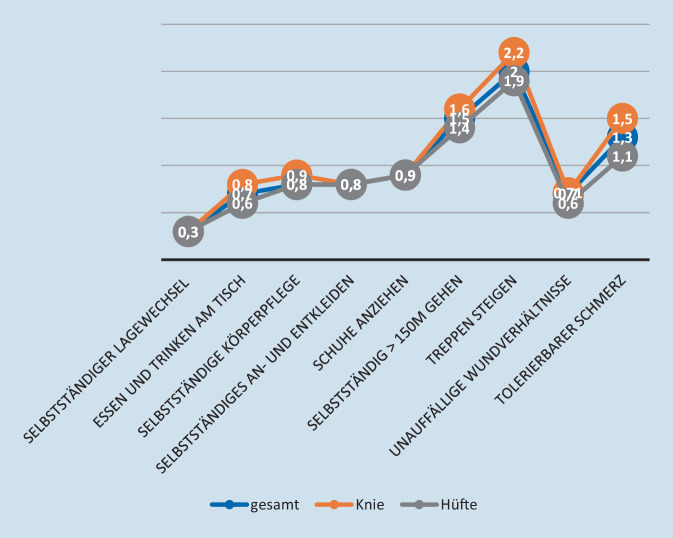

Eine detaillierte Übersicht zu den Quoten, mit denen die Kriterien erreicht werden, und wann diese erreicht werden, finden Sie in Tab. 2 und Abb. 1.

**Table 2:** 

		Gesamt	Knie	Hüfte
*Selbstständiger Lagewechsel (Liegen, Sitzen, Stehen)?*	Ja, erreicht (*n*)	99,9 % (1777)	99,9 % (879)	99,9 % (896)
Zeit bis Erfüllung (in Tagen)	Mittelwert (SD)	0,3 (0,8)	0,3 (0,9)	0,3 (0,6)
	Median (Q1; Q3)	0 (0; 0)	0 (0; 0)	0 (0; 1)
	Spannweite	0–20	0–20	0–7
*Selbstständiges Essen und Trinken am Tisch*	Ja, erreicht (*n*)	97,1 % (1722)	96,7 % (848)	97,5 % (872)
Zeit bis Erfüllung (in Tagen)	Mittelwert (SD)	0,7 (0,9)	0,8 (1,1)	0,6 (0,7)
	Median (Q1;Q3)	1 (0; 1)	1 (0; 1)	1 (0; 1)
	Spannweite	0–20	0–20	0–6
*Selbstständige Körperpflege*	Ja, erreicht (*n*)	99,2 % (1761)	99,5 % (875)	98,8 % (884)
Zeit bis Erfüllung (in Tagen)	Mittelwert (SD)	0,8 (0,8)	0,9 (1,0)	0,8 (0,7)
	Median (Q1; Q3)	1 (0; 1)	1 (0; 1)	1 (0; 1)
	Spannweite	0–21	0–21	0–7
*Selbstständiges An- und Entkleiden*	Ja, erreicht (*n*)	99,5 % (1768)	99,5 % (874)	99,6 % (892)
Zeit bis Erfüllung (in Tagen)	Mittelwert (SD)	0,8 (0,8)	0,8 (1,0)	0,8 (0,7)
	Median (Q1; Q3)	1 (0; 1)	1 (0; 1)	1 (0; 1)
	Spannweite	0–21	0–21	0–6
*Schuhe anziehen*	Ja, erreicht (*n*)	98,7 % (1709)	98,9 % (835)	98,5 % (872)
Zeit bis Erfüllung (in Tagen)	Mittelwert (SD)	0,9 (1,3)	0,9 (1,1)	0,9 (1,3)
	Median (Q1; Q3)	1 (0; 1)	1 (0; 1)	1 (0; 1)
	Spannweite	0–29	0–20	0–29
*Selbstständig >* *150m gehen*	Ja, erreicht (*n*)	96,1 % (1708)	95,7 (841)	96,7 % (866)
Zeit bis Erfüllung (in Tagen)	Mittelwert (SD)	1,5 (1,3)	1,6 (1,6)	1,4 (0,9)
	Median (Q1; Q3)	1 (1; 2)	1 (1; 2)	1 (1; 2)
	Spannweite	0–23	0–23	0–11
*Treppen steigen*	Ja, erreicht (*n*)	95,4 % (1696)	95,1 % (873)	95,9 % (857)
Zeit bis Erfüllung (in Tagen)	Mittelwert (SD)	2,0 (1,4)	2,2 (1,7)	1,9 (1,1)
	Median (Q1; Q3)	2 (1; 2)	2 (1; 3)	2 (1; 3)
	Spannweite	0–23	0–23	0–11
*Unauffällige Wundverhältnisse*	Ja, erreicht (*n*)	98,9 % (1760)	98,1 % (862)	99,8 % (896)
Zeit bis Erfüllung (in Tagen)	Mittelwert (SD)	0,7 (1,5)	0,71 (1,7)	0,6 (1,2)
	Median (Q1; Q3)	0 (0; 1)	0 (0; 1)	0 (0; 1)
	Spannweite	0–23	0–23	0–22
*Tolerierbarer Schmerz*	Ja, erreicht (*n*)	95,2 % (1691)	93,0 % (816)	97,2 % (873)
Zeit bis Erfüllung (in Tagen)	Mittelwert (SD)	1,3 (1,5)	1,5 (1,4)	1,1 (1,5)
	Median (Q1; Q3)	1 (0; 2)	1 (0; 2)	1 (0; 2)
	Spannweite	0–27	0–20	0–27

## Die nicht erreichten Entlasskriterien

Bei 15 % (*n* = 262) der Patienten wird mindestens ein Entlasskriterium im stationären Aufenthalt nicht erreicht, meist ein einzelnes Kriterium (60 %; *n* = 158). Mit zunehmender Anzahl der nicht erreichten Kriterien nimmt auch der Prozentsatz der betroffenen Patienten ab. Eine Übersicht zu den Ergebnissen zeigt Tab. 3.

**Table 3:** 

	Gesamt	Knie	Hüfte
1 Kriterium nicht erfüllt (*n*)	60,3 % (158)	63,9 % (106)	53,7 % (51)
2 Kriterien nicht erfüllt (*n*)	24,4 % (64)	22,3 % (37)	28,4 % (27)
3 Kriterien nicht erfüllt (*n*)	7,3 % (19)	6,0 % (10)	9,5 % (9)
4 Kriterien nicht erfüllt (*n*)	4,6 % (12)	5,4 % (9)	3,2 % (3)
5 Kriterien nicht erfüllt (*n*)	1,5 % (4)	0,6 % (1)	3,2 % (3)
6 Kriterien nicht erfüllt (*n*)	0,4 % (1)	0,6 % (1)	0
7 Kriterien nicht erfüllt (*n*)	1,1 % (3)	1,2 % (2)	1,1 % (1)
8 Kriterien nicht erfüllt (*n*)	0,4 % (1)	0	1,1 % (1)

**Figure 2:**
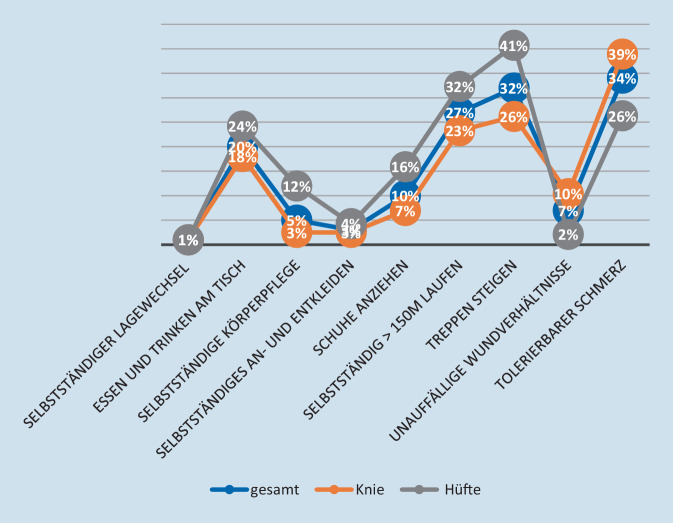

In der Gruppe EKn (14,7 %, *n* = 262) sind die verschiedenen Kriterien zu sehr unterschiedlichen Prozentsätzen nicht erreicht worden. Mit mehr als 20 % Anteil (*n* > 52) sind folgende Kriterien besonders oft beteiligt: Essen und Trinken am Tisch (Hüfte), selbstständig > 150 m gehen (Knie und Hüfte), Treppen steigen (Knie und Hüfte), tolerierbarer Schmerz (Knie und Hüfte) (Abb. 2).

Aus den Freitextkommentaren zu den Gründen für das nicht Erreichen der Entlasskriterien kann abgelesen werden, dass diese sehr unterschiedlich sind und teilweise miteinander zusammenhängen, z. B. „kein Treppen steigen wegen zu starker Schmerzen“. Teilweise ist die Durchführung des Kriteriums tatsächlich noch nicht möglich, z. B. das selbstständige Gehen > 150 m wegen „Schwäche“, „seit 5 Jahren kaum mehr gegangen“, oder „Notwendigkeit zur Entlastung wegen periprothetischer Fraktur“. Ein Teil der Patienten konnte die geforderten Kriterien auch vor der Operation schon nicht erfüllen, z. B. „Pat. benötigt auch zuvor Hilfe beim Waschen“. Teilweise sind aber auch die Rahmenbedingungen Ursache für das Nichterreichen, z. B. „Stühle zu niedrig, Zimmer zu eng“ oder „bei Adipositas per magna passte der Patient nicht an den Tisch“. Teilweise wird der Patient nicht ausreichend über das Kriterium informiert und zur Umsetzung aufgefordert, z. B. „Schwester stellt Essen ans Bett“, oder er lehnt die Umsetzung ab, z. B. „Pat. wollte nicht am Tisch sitzen“, „Findet es im Bett bequemer“.

## Diskussion

Würden die sogenannten Entlasskriterien, wie für Dänemark beschrieben [8], stringent umgesetzt, wären nach den hier vorliegenden Daten für Patienten nach Implantation einer Hüft- oder Knieendoprothese im Rahmen eines optimierten Behandlungsprozesses auch in Deutschland international übliche stationäre Aufenthaltszeiten [6–9] Realität. Tatsächlich aber verbleiben die Patienten mehr als doppelt so lange im Krankenhaus, wie es für das Erreichen der Entlasskriterien notwendig ist. Gleichzeitig werden von einer nicht unerheblich großen Gruppe nicht alle zuvor definierten Entlasskriterien erreicht. Diese Gruppe wird im Mittel trotzdem kaum später aus dem Krankenhaus entlassen.

Beide Tatsachen weisen darauf hin, dass Entlasskriterien in Deutschland als relative Kriterien eingesetzt werden, die im Sinne einer Zieldefinition erreicht werden sollen, aber nicht erreicht werden müssen. Unterstützt wird dieses Vorgehen eventuell dadurch, dass kein Set allgemein anerkannter und vorgeschriebener Entlasskriterien existiert. Wie beschrieben, gibt es zwar grundsätzliche Übereinstimmungen, aber in Detail und im Differenzierungsgrad bestehen Unterschiede. So wird in [17] zusätzlich zum selbstständigen An- und Entkleiden speziell das Anziehen der Schuhe gefordert, in [15–17] das Treppen steigen und in [14] das Gehen zur Toilette. Auch die geforderte Gehstrecke differiert mit 45 m [15], 100 m [16] oder 200 m [12]. Teilweise sind die Kriterien auch nicht mit objektivierbaren Schwellenwerten hinterlegt (Gehen an Unterarmgehstützen [8], Treppen steigen [15]). Relativiert werden Entlasskriterien auch dadurch, dass sie teilweise schon vorher nicht erreichbar waren, im Einzelfall durch äußere Rahmenbedingungen kaum zu erreichen sind, von den beteiligten Berufsgruppen nicht entsprechend eingefordert oder vom Patienten abgelehnt werden.

An welchen Kriterien sich die Entlassung in Deutschland tatsächlich orientiert, bleibt offen. Da Deutschland weiterhin über weit mehr stationäre Betten verfügt als andere Länder (Krankenhausbetten je 1000 Einwohner 2019, Deutschland: 7,91; Dänemark: 2,59 [21]) ist der Belegungsdruck weit geringer als in fast allen anderen Ländern der Welt. Dies könnte dazu führen, dass die Verantwortlichen den Patienten weitere Tage stationären Aufenthaltes zugestehen, obwohl die Entlasskriterien bereits erreicht sind. Wir beobachten in der Praxis, dass diese Tage von Patienten teils aktiv eingefordert werden, auch um die Zeit bis zur Aufnahme in eine stationäre Rehabilitationsmaßnahme zu überbrücken. Dies ist eine Besonderheit Deutschlands, da nur hier für alle Patienten ein Anspruch auf eine Anschlussheilbehandlung besteht.

Die Tatsache der großen Anzahl verfügbarer Betten könnte auch die Frage der Wirtschaftlichkeit einer früheren Entlassung beeinflussen. Nöth et al. [22] berechnen zwar für ein Krankenhaus mit 1120 Fällen und einer Reduktion der Aufenthaltszeit von 7 auf 5 Tage, eine theoretisch mögliche Fallzahlsteigerung um 448 Fällen. Würden lediglich 25 % davon umgesetzt, wäre dies mit einer Umsatzsteigerung von 684.649 € verbunden. Sie merken jedoch an, dass dies nur bei entsprechender Operationskapazität und entsprechendem Personal realisiert werden kann. Zudem muss für die Realisierung zusätzlicher Umsätze die Nachfrage nach zusätzlichen Operationen bestehen. Hier könnte sich die absehbare Alterung unserer Gesellschaft auswirken, die mit einer Projektion bis 2050 zu 43 % mehr Kniegelenksimplantationen [23] und bis 2060 zu 62 % mehr Hüftgelenksimplantationen [24] führen könnte. Die gesellschaftlichen Entwicklungen sind jedoch auch direkt verbunden mit dem immer drängender werdenden Problem des Fachkräftemangels [25]. Dieser könnte über die Limitierung der betreibbaren Krankenausbetten Druck auf die Umsetzung kürzerer stationärer Aufenthaltszeiten erhöhen. Ganz aktuell wird in der Corona-Pandemie deutlich, wie wertvoll und limitiert Ressourcen zur stationären Behandlung sind, sodass die Diskussion um kürzere stationäre Aufenthaltszeiten intensiver werden könnte [26]. Vor dem Hintergrund der Pandemie könnte auch das lang bekannte, aber jetzt ganz aktuell sehr bewusst gewordene Problem einer möglichen Infizierung im Krankenhaus neu bewertet werden. Auch aus dieser Sicht sind verkürzte stationäre Aufenthaltszeiten positiv zu bewerten [26].

Neben der bereits diskutierten Frage der Realisierbarkeit errechneter finanzieller Vorteile einer Verkürzung stationärer Aufenthalte, kann auf der anderen Seite auch das Vermeiden von finanziellen Nachteilen durch das in Deutschland bestehende DRG-System mit vorgegebenen unteren Grenzverweildauern und drohenden Kurzliegerabschlägen bei zu frühen Entlassungen Einfluss auf die stationären Aufenthaltszeiten ausüben [27]. Sind diese Abschläge der Grund für die Divergenz zwischen Entlassbarkeit und Entlassung, könnte die Neufassung der gesetzlichen Rahmenbedingungen zu den Qualitätsverträgen (§ 110a SGB V) eine Chance bieten. Optimierte Versorgungen auf Basis der evidenzbasierten Empfehlungen der ERAS-Society [1, 2], wie die des PROMISE-Projektes, sind ohne Weiteres geeignet, die verbindlichen Rahmenvorgaben zum Abschluss eines solchen Vertrages [28] zu erfüllen. So wäre es möglich, zu vereinbaren, dass bei messbarer Qualitätssicherung durch das Krankenhaus [29] die Abschläge durch die Krankenkasse ersetzt werden. Die verbindlich begleitende Evaluation der Qualitätsverträge durch das Institut für Qualitätssicherung und Transparenz im Gesundheitswesen (IQTIG) könnte die Wirkung einer solchen Vereinbarung auf das Verhältnis von Entlassbarkeit und Entlassung ermitteln.

Die Stärken der Studie sehen wir in einem prospektiven, multizentrischen, sektorenübergreifenden Design, einer sehr großen Anzahl von Probanden, einer sehr breiten Datenerhebung zu zahlreichen Zeitpunkten, einer überdurchschnittlich langen Nachbeobachtungszeit und einer unabhängigen und externen Evaluation. Es wurden dabei Daten erhoben, die für Deutschland bisher nicht zu Verfügung standen. Einschränkend muss aber festgehalten werden, dass das Verhältnis von Entlassbarkeit und tatsächlicher Entlassung nicht der primäre Fokus der Studie war. Daher fehlen entscheidende Informationen und der tatsächliche Trigger für eine Entlassung bleibt weiterhin unbekannt. Allerdings können oder müssen wir nun davon ausgehen, dass aktuell das Erreichen zuvor definierter Entlasskriterien im Entlassmanagement nicht die zu erwartende Rolle spielt.

## Fazit für die Praxis

- Bei stringenter Umsetzung von Entlasskriterien können in Deutschland im Rahmen eines optimierten Behandlungsprozesses für Patienten nach Implantation von Hüft- und Knieendoprothese international übliche stationäre Aufenthaltszeiten realisiert werden.
- Aktuell beträgt die stationäre Aufenthaltszeit mehr als das Doppelte der durch Entlasskriterien definierten Zeitspanne.
- Das Erreichen der Entlasskriterien ist nicht der primäre Trigger für eine Entlassung.
- Was die Aufenthaltszeit definiert, bleibt offen.

## Abkürzungen

ASA: American Society of Anesthesiologists
DRG: Diagnosis Related Groups
EK: Entlasskriterien
EKe: Entlasskriterien erreicht
EKn: Entlasskriterien nicht erreicht
ERAS: Enhanced Recovery after Surgery
IQTIG: Institut für Qualitätssicherung und Transparenz im Gesundheitswesen
ISAR: Identification of Seniors at risk
MW: Mittelwert
NRS: Numerische Rating-Skala
SGB: Sozialgesetzbuch
TEP: Totalendoprothese
